# A glycosaminoglycan microarray identifies the binding of SARS‐CoV‐2 spike protein to chondroitin sulfate E

**DOI:** 10.1002/1873-3468.14173

**Published:** 2021-08-17

**Authors:** Tomoko Watanabe, Ko Takeda, Keiko Hiemori, Toshikazu Minamisawa, Hiroaki Tateno

**Affiliations:** ^1^ Cellular and Molecular Biotechnology Research Institute National Institute of Advanced Industrial Science and Technology (AIST) Tsukuba Japan; ^2^ Central Research Laboratory Seikagaku Corporation Higashiyamato‐shi Japan

**Keywords:** chondroitin sulfate, glycosaminoglycan, heparan sulfate, microarray, S protein, SARS‐CoV‐2

## Abstract

Heparan sulfate (HS), a sulfated glycosaminoglycan (GAG), was reported to be a necessary host attachment factor that promotes SARS‐CoV‐2 infection. In this study, we developed GAG microarrays based on fluorescence detection for high‐sensitivity screening of the GAG‐binding specificity of proteins and applied it for the analysis of SARS‐CoV‐2 spike (S) protein. Among the 20 distinct GAGs, the S protein bound not only to heparin (HEP)/HS but also to chondroitin sulfate E (CSE) in a concentration‐dependent manner. We then analyzed the specificity of each subunit of the S protein. While the S1 subunit showed exclusive binding to HEP, the S2 subunit also bound to CSE and HEP/HS. CSE might act as an alternative attachment factor for HS in SARS‐CoV‐2 infection.

## Graphical abstract

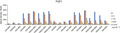

## Abbreviations

**ACE2**, angiotensin converting enzyme 2

**CH**, chondroitin

**COVID‐19**, coronavirus disease 2019

**CS**, chondroitin sulfate

**CSE**, chondroitin sulfate E

**DS**, dermatan sulfate

**GAG**, glycosaminoglycan

**HA**, hyaluronic acid

**HEP**, heparin

**HPN**, heparosan

**HS**, heparan sulfate

**KS**, keratan sulfate

**RBD**, receptor‐binding domain

**S protein**, spike protein

**SARS‐CoV‐2**, severe acute respiratory syndrome coronavirus 2

A novel coronavirus, severe acute respiratory syndrome coronavirus 2 ‘(SARS‐CoV‐2)’, which caused coronavirus disease 2019 (COVID‐19), was first discovered in December 2019, in Wuhan, Hubei province, China [1]. COVID‐19 is an infectious disease that manifests as cough, fever, and severe pneumonia. As of February 26, 2021, the number of infected people worldwide was approximately 112.20 million, and the death toll was approximately 2.49 million [2].

SARS‐CoV‐2 entry into host cells is mediated by the transmembrane spike (S) protein, which forms homotrimers that protrude from the viral surface. The S protein consists of two functional subunits that are responsible for binding to the host cell receptor (S1 subunit) and fusion of the viral and cellular membranes (S2 subunit) [3, 4]. The S protein interacts with both cellular heparan sulfate (HS) glycosaminoglycan (GAG) and angiotensin converting enzyme 2 (ACE2) *via* its receptor‐binding domain (RBD) in the S1 subunit to enter target cells [5]. After RBD–ACE2 binding, the S protein was cleaved and removed the outer S1 subunit by host proteases, thus exposing the inner S2 subunit [6]. The S2 subunit binds to host cell membranes, thereby enabling the virus to invade host cells and initiate replication [6]. The binding capacity of the SARS‐CoV‐2 S protein to GAGs other than HS has also been reported [7, 8], suggesting the importance of other types of GAGs as attachment factors for SARS‐CoV‐2.

Proteoglycans exist in the extracellular matrix or on the cell surface and consist of a core protein and one or more covalently attached GAG chains, which are linear polysaccharides containing disaccharide building blocks that consist of an amino sugar (glucosamine that is N‐acetylated or N‐sulfated or N‐acetylgalactosamine) and a uronic acid (glucuronic acid or iduronic acid) or galactose [9]. On the basis of their disaccharide compositions, GAGs are classified into four groups: heparin (HEP)/HS, chondroitin sulfate (CS)/dermatan sulfate (DS), keratan sulfate (KS), and hyaluronic acid (HA). GAG chains have a large number of sulfate groups and are highly negatively charged, except for HA.

In this study, we developed GAG microarrays for high‐throughput screening of the GAG‐binding specificity of proteins. GAGs from natural sources, such as HEP, and its desulfated forms, HS, CS (CSA, CSB [DS], CSC, CSD, CSE), chondroitin (CH), heparosan (HPN), and HA, with a total of 20 GAGs, were conjugated with bovine serum albumin (BSA) and immobilized on a microarray platform. The efficacy of the developed GAGs was first validated with a well‐known HEP/HS‐binding protein, fibroblast growth factor (FGF) 1. We then applied the microarrays to screen for GAGs that are recognized by the SARS‐CoV‐2 S protein, which consists of S1 and S2 subunits. Whereas the S1 subunit showed exclusive binding to HEP, the S2 subunit bound to HEP, HS, and CSE. The binding activity of the S protein to HS‐BSA could be inhibited by CSE and HEP/HS. We therefore conclude that CSE might act as an alternative host attachment factor for HS, which promotes SARS‐CoV‐2 infection of various target cells. The S2 subunit might play a substantial role in the interaction between S protein and GAGs.

## Materials and methods

### GAG

Sources of GAGs used in this study are summarized in Table S1. CSA, CSB (DS), CSC, CSD, CSE, and HA were obtained from Seikagaku Co. (Tokyo, Japan). HEP was obtained from Fujifilm Wako Pure Chemical Co. (Osaka, Japan). HS was prepared following a previously described method, but with minor modifications [10]. CH was prepared from the *Escherichia coli* K12 strain, as previously described [11]. HPN was prepared from *E. coli* K5 strain, as previously described [12].

### Disaccharide composition analysis

GAGs were characterized by conventional disaccharide composition analysis [13]. CS (CSA, CSB [DS], CSC, CSD, or CSE), CH, or HA was dissolved at 0.5 mg/mL with 20 mm Tris/HCl (pH 8.0) containing chondroitinase ABC I, chondroitinase ABC II, and chondroitinase AC II. HS, HEP, desulfated heparin, or HPN was dissolved at 0.5 mg/mL with 20 mm sodium acetate (pH 7.0) and 3 mm calcium acetate containing heparitinase I, heparitinase II, and heparinase (Seikagaku Corp., Tokyo, Japan). After incubation overnight at 37 °C, the enzymes were removed using a Nanosep 10K centrifugal ultrafiltration filter (Pall Corporation, Ann Arbor, MI, USA). The disaccharide composition of GAGs was determined by reversed‐phase ion‐pair high‐performance liquid chromatography (Dionex UltiMate 3000, Thermo Fisher Scientific, Waltham, MA, USA) equipped with a Senshu Pak Docosil column (4.6 × 150 mm; Senshu Scientific Co., Tokyo, Japan) and coupled with postcolumn labeling, as previously described [13]. Each disaccharide peak was identified by reference to the retention times of the CS unsaturated disaccharide standards (Seikagaku Co., Tokyo, Japan). The molar concentrations of each CS or HS disaccharide in the sample solutions were determined on the basis of a calculation of the area ratio between the sample and the disaccharide standards. Each disaccharide composition was calculated from the ratio of the disaccharide molar concentration to the total disaccharide molar concentration.

### Preparation of GAG–BSA conjugates

To introduce biotin to carboxyl groups of GAG, GAG (33.3 mg/mL) dissolved in 25 mm sodium borate buffer (pH 9.5) was mixed with 40 mm aminobiotin and 40 mm 4‐(4,6‐dimethoxy‐1,3,5‐triazin‐2‐yl)‐4‐methylmorpholinium chloride (Wako Pure Chemical Industries) at the molar ratio of 10 : 1 : 2 and reacted at room temperature overnight. After the reaction, biotinylated GAG was precipitated by ethanol and was lyophilized.

Biotinylated GAG was conjugated with BSA as previously described, but with some modifications [14]. Aminoalkanethiol (40 mg/mL) dissolved in deionized water was mixed with 50 mg/mL biotinylated GAG and sodium cyanoborohydride solution (Tokyo Chemical Industry Co., Ltd., Tokyo, Japan) and reacted under dark conditions at 43 °C for 16 h. The SH‐modified GAG that was generated at the reducing end was then purified by ethanol precipitation, lyophilized, and stored at −20 °C under dark conditions. BSA dissolved on Dulbecco's phosphate‐buffered saline (10 mg/mL, Thermo Fisher Scientific, MA, USA) was incubated with 1/10 of a bifunctional linker, *N*‐(11‐maleimidoundecanoyloxy) succinimide (Dojindo Molecular Technologies., Inc., Kumamoto, Japan), under dark conditions at room temperature for 1 h. The generated maleimide‐conjugated BSA was purified by removing insoluble materials with a 0.22 µm polyvinylidene difluoride membrane, diluted with phosphate‐buffered saline concentrated several times with AmiconUltra50K (Millipore, MA, USA), and stored under dark conditions at −20°C. Maleimide‐modified BSA (1 mg/mL) was incubated with SH‐modified GAG (13.8 mg/mL) in the dark at room temperature for 16 h. Cysteine at the final concentration of 0.5 mm was added into the reaction mixture and then incubated at room temperature for 10 min to quench unreacted maleimide conjugated to BSA. Unreacted maleimide‐modified GAG was removed by 2 M NaCl dilution and then concentrated three times with AmiconUltra50K or 100K (Millipore, MA, USA). The generated GAG‐BSA conjugate was diluted in 20 mm ammonium formate and then concentrated three times with AmiconUltra50K or 100K (Millipore, MA, USA). Next, the GAG‐BSA conjugate was diluted with deionized water and then concentrated twice with AmiconUltra50K or 100K (Millipore, MA, USA). Finally, the generated GAG‐BSA conjugate was analyzed by size‐exclusion chromatography on YMC‐Pack Diol‐300 (YMC Co., Ltd., Kyoto, Japan).

### GAG microarray production

GAG microarrays were produced as previously described [15]. The GAG‐BSA conjugates were dissolved in a spotting solution (Matsunami Glass Ind., Ltd, Osaka, Japan). After insoluble particles were removed *via* filtration through a 0.22‐μm pore size filter, the GAG‐BSA conjugates were spotted in triplicate on a microarray‐grade epoxy‐coated glass slide (Schott AG, Mainz, Germany) attached to a silicone rubber sheet with seven chambers using a noncontact microarray printing robot (MicroSys 4000; Genomic Solutions Inc., MI, USA) with a spot diameter size of 500 μm. The glass slide was incubated in a humidity‐controlled incubator at 25 °C for 3 h to enable immobilization. After incubation, excess amounts of nonimmobilized materials were washed away with the probing buffer (25 mm Tris/HCl, pH 7.4 containing 0.8% NaCl, 1% (v/v) Triton X‐100, 1 mm MnCl2, 1 mm CaCl_2_) and blocked with 100 μL TBS (25 mm Tris/HCl, pH 7.4 containing 0.8 NaCl) containing 1% BSA at 20°C for 1 h.

### Preparation of glycan‐binding proteins

Recombinant FGF1 (16–155 amino acids) was purchased from R&D Systems. Recombinant SARS‐CoV‐2 S protein (16–1210 amino acids, monomeric form) and SARS‐CoV‐2 S1 subunit (16–675 amino acids) expressed in HEK293 cells were purchased from Bio‐Serv (Flemington, NJ, USA). The recombinant SARS‐CoV‐2 S2 subunit (697–1213 amino acids) expressed in HEK293 cells was purchased from RayBiotech (Peachtree Corners, GA, USA). The carbohydrate recognition domain of galectin‐3 (108‐250 aa, P17931) and galectin‐7 (8‐136 aa, P47929) was cloned into pET27b expression vector and recombinantly expressed in *E. coli* [16]. Purification was performed on lactose‐Sepharose CL‐4B. Glycan‐binding specificity was evaluated by glycoconjugate microarray [15].

### GAG microarray analysis

Proteins were fluorescently labeled with Cy3 Mono‐Reactive dye (Cytiva, Tokyo, Japan), and excess Cy3 was removed with Sephadex G‐25 desalting columns (Cytiva). Cy3‐labeled proteins diluted in 25 mm HEPES buffer (pH 7.5) containing 150 mm NaCl and 1% Triton X‐100 were applied to each chamber of the glass slides (80 µL/well) and were incubated at 20 °C for 3 h, after which the fluorescent images were acquired immediately using a Bio‐Rex scan 200 fluorescence scanner (Rexxam Co. Ltd., Kagawa, Japan). The net intensity value for each spot was determined by signal intensity minus the background value. Data are the average ± SD of triplicate spots.

## Results

### Generation of GAG microarrays

**Fig. 1:**
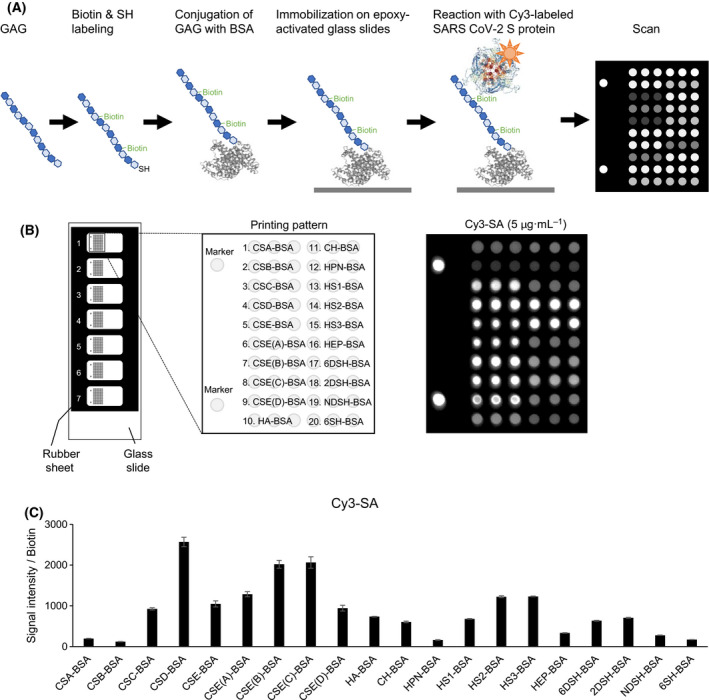
Development of GAG microarrays. (A) Schematic illustration of the generation and analysis of GAG microarrays. (B) Printing pattern of GAG microarrays (*left*) and evaluation of immobilized GAGs by Cy3–SA (*right*). (C) GAG microarrays incubated with Cy3–SA and detected by the scanner without washing. Signal intensity divided by the amount of biotin to indicate the amount of the immobilized GAGs. Data are the average ± SD of triplicate spots.

GAGs from natural sources with or without modifications were conjugated on BSA (GAG‐BSA), which were subsequently covalently immobilized on epoxy‐activated microarray glass slides (Fig. 1A). A series of GAGs, including CS (CSA, CSB [DS], CSC, CSD, CSE), HA, CH, HPN, HS, and HEP, were used (Table S1). For CSE, HS, and HEP, different MWs of CSE (A: 46.7 kDa, B: 26 kDa, C: 35.1 kDa, D: 17.5 kDa) and HS (HS1: 11.6 kDa, HS2: 26.7 kDa, HS3: 32.6 kDa) and different sulphation patterns of HEP (HEP, 6DSH [6‐O‐desulfated], 2DSH [2‐O‐desulfated], NDSH [N‐desulfated], and 6SH [only 6‐O‐sulfated]) were prepared. The disaccharide composition of the GAGs was evaluated by high‐performance liquid chromatography in adherence to disaccharide standards (Table S1). GAGs were labeled with SH groups at the reducing end and were conjugated with BSA with the bifunctional linker, *N*‐(11‐maleimidoundecanoyloxy) succinimide, through the SH groups of GAGs and the amino groups of BSA. Biotin was introduced into the GAGs to quantify the amounts of GAGs that were immobilized on the microarray chips.

GAG‐BSA was spotted on microarray chips with 0.5‐mm spot diameter size using a microarray spotter, as shown in Fig. 1B. The amount of GAG–BSA immobilized on the microarray chips was evaluated by Cy3‐labeled streptavidin (SA; Fig. 1B). GAG microarrays were incubated with Cy3‐SA (5 μg/mL) and scanned using the fluorescence scanner at equilibrium without washing. The fluorescence signal intensity of SA was divided by the amount of biotin conjugated on each GAG to determine the amounts of GAGs that were immobilized on the microarray chips (Fig. 1C). To immobilize similar amounts of GAGs on the microarray chips, the spotting concentration of GAG–BSA was adjusted so that the signal intensity/biotin is in the range between 100 and 3000 (Fig. 1C and Table S1).

### Validation of the efficacy of GAG microarrays by FGF1

**Fig. 2:**
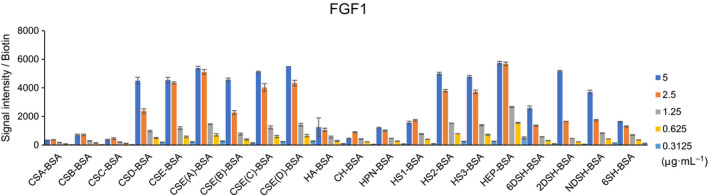
Screening of GAG‐binding specificity of FGF1. GAG microarrays were incubated in varying concentrations of FGF1 (0.3125–5 μg/mL) and were detected by the scanner without washing. Signal intensity was divided by the amount of biotin. Data are the average ± SD of triplicate spots.

The efficacy of the developed GAG microarrays was first validated by FGF1, which is a member of a large family of growth factors that are involved in cell proliferation, development, and tumor angiogenesis and serves as a representative protein with binding specificity to sulfated GAGs [15]. Cy3‐labeled FGF1 (0.3–5 μg/mL) was incubated with GAG microarrays. The obtained fluorescence signal intensity was divided by the amount of biotin on each GAG. As shown in Fig. 2, FGF1 bound strongly to HEP‐BSA, HS (HS2 and HS3)‐BSA, CSE‐BSA, and CSD‐BSA in a concentration‐dependent manner. Binding of FGF1 to HEP‐BSA was decreased by desulphation at 6‐O‐, 2‐O‐, and N‐positions (6DSH, 2DSH, NDSH, and 6SH), which was consistent with previous reports [15, 17]. In contrast, only a weak signal was observed for CSA–BSA, CSB (DS)–BSA, CSC–BSA, and unsulfated GAGs‐BSA such as HA‐BSA, CH‐BSA, and HPN‐BSA. These results demonstrate the efficacy of the developed GAG microarrays for screening GAG‐binding specificity of proteins in a high‐sensitivity and high‐throughput manner.

### Screening of the GAG‐binding specificity of SARS‐CoV‐2 S protein

**Fig. 3:**
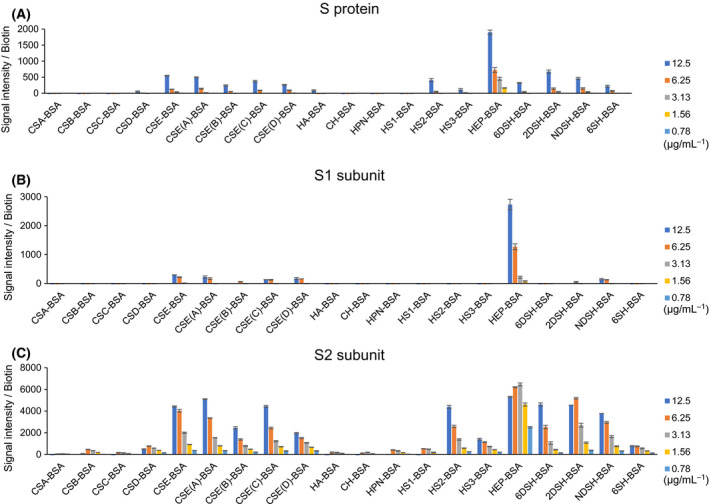
Screening of the GAG‐binding specificity of SARS‐CoV‐2 S protein. GAG microarrays were incubated in varying concentrations of SARS‐CoV‐2 S protein, S1 subunit, and S2 subunit (0.78–12.5 μg/mL) and were detected by the scanner without washing. Signal intensity was divided by the amount of biotin. Data are the average ± SD of triplicate spots.

The GAG microarrays were then applied to screen for GAGs recognized by the SARS‐CoV‐2 S protein, which consists of S1 and S2 subunits [3, 4]. The binding capacity of the SARS‐CoV‐2 S protein to various GAGs has been analyzed using various binding analyses such as surface plasmon resonance (SPR), pulldown assay, flow cytometry, cell‐based ELISA, and viral entry assays (Table S2) [5, 8, 18, 19, 20, 21, 22, 23, 24, 25, 26]. The SARS‐CoV‐2 S protein (monomeric form) showed the highest signal intensity to HEP‐BSA (Fig. 3A). The binding of the S protein to HEP‐BSA was significantly decreased by desulphation at 6‐O‐, 2‐O‐, and N‐positions (6DSH‐BSA, 2DSH‐BSA, NDSH‐BSA, and 6SH‐BSA). The S protein also showed a significant but weaker binding to CSE and its fragments (CSE [A], CSE [B], CSE [C]), CSE [D], and HS2 (Fig. 3A).

To determine which subunit of the SARS‐CoV‐2 S protein is involved in GAG binding, the binding specificity of each of the S1 and S2 subunits was then analyzed. Interestingly, the S1 subunit showed almost exclusive binding to HEP (Fig. 3B). The binding activity of S1 to HEP was abrogated by desulphation at any of the sulphation positions (6DSH, 2DSH, NDSH, and 6SH), which indicated that all of the sulphation positions at 6‐O‐, 2‐O‐, and N‐positions substantially contribute to S1 binding to HEP. The S2 subunit also showed the highest signal to HEP at a lower concentration (0.78 μg/mL) than that of the S1 subunit (>3.13 μg/mL), which indicated that the S2 subunit might show higher binding activity to HEP than the S1 subunit (Fig. 3C). S2 binding to HEP was decreased by desulphation at any position (6DSH, 2DSH, NDSH, and 6SH). To exclude the possibility that any proteins artificially absorb to sulfated GAGs such as HEP‐BSA, we analyzed galectin‐3 and galectin‐7, which were previously reported to bind to unsulfated GAGs such as CH [27]. Consistently, galectin‐3 and galectin‐7 bound specifically to CH‐BSA, but not to sulfated GAGs (Fig. S1), demonstrating the specific interaction between proteins and GAG‐BSA. We concluded that the S1 subunit binds exclusively to HEP, whereas the S2 subunit binds not only to HEP but also to CSE and HS.

### Inhibitory activity of free GAG chains on the binding of the SARS‐CoV‐2 S protein to GAG‐BSA

**Fig. 4:**
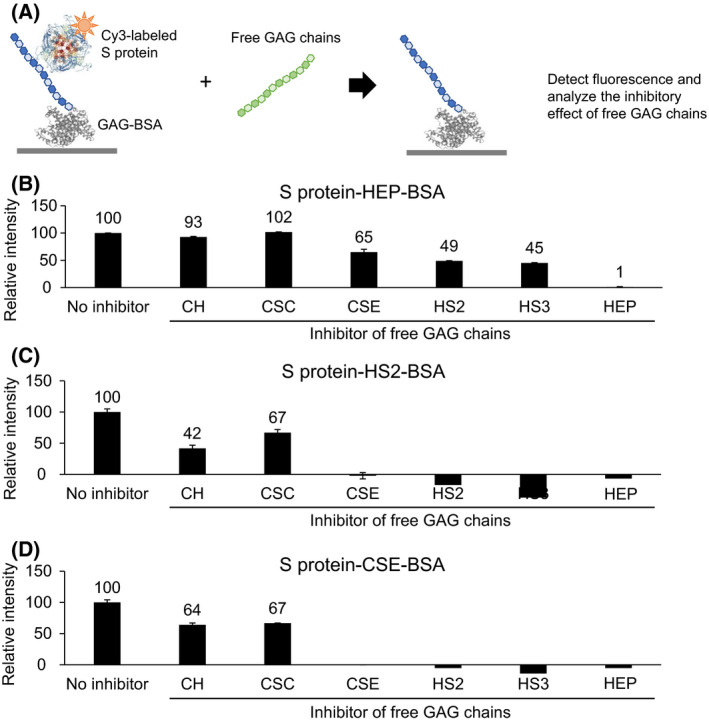
Inhibitory activity of free GAG chains on the interaction between SARS‐CoV‐2 S protein and GAG‐BSA. SARS‐CoV‐2 S protein was incubated with GAG microarrays in the presence or absence of 1 mg/mL of free GAG chains (A). The relative fluorescence intensity of S protein to HEP–BSA (B), HS2–BSA (C), and CSE–BSA (D) was compared as the ‘no inhibitor’ signal at 100.

Finally, the inhibitory activity of free GAG chains on the binding of the SARS‐CoV‐2 S protein to HEP–BSA, HS2–BSA, and CSE–BSA was analyzed. The SARS‐CoV‐2 S protein was incubated with GAG microarrays in the presence or absence of free GAG chains (1 μg/mL; Table S1), and the inhibitory activity of free GAG chains was compared (Fig. 4). Free HEP completely inhibited the binding of the SARS‐CoV‐2 S protein to HEP–BSA, HS2–BSA, and CSE–BSA. Not only free HEP but also free HS (HS2 and HS3) and free CSE abrogated the binding of the S protein to HS2–BSA as well as CSE–BSA (Fig. 4). Since the S protein showed no significant binding to other CSs such as CSA, CSB, CSC, or CSD, disulphation at 4‐O‐ and 6‐O‐ positions in the GalNAc residue is essential for S protein binding to CSE.

## Discussion

GAGs are often utilized by viral pathogens as attachment factors, which facilitates their initial interaction with host cells, including herpes simplex virus (HSV) and different coronaviruses (SARS‐CoV‐1 and MERS‐CoV) [28, 29, 30]. Clausen *et al*. provided clear evidence that HS is an essential host attachment factor that promotes SARS‐CoV‐2 infection in various target cells [5]. The RBD of the S1 subunit of SARS‐CoV‐2 binds to HEP/HS *via* a docking site composed of positively charged amino acid residues that are aligned in a subdomain of the RBD, which is separate from the site that is involved in ACE2 binding. The addition of HEP and its derivatives was demonstrated to inhibit SARS‐CoV‐2 infection [5]. The binding capacity of the SARS‐CoV‐2 S protein to various GAGs has also been reported using various binding analyses such as SPR, pulldown assay, flow cytometry, cell‐based ELISA, and viral entry assays (Table S2) [5, 8, 18, 19, 20, 21, 22, 23, 24, 25, 26].

In this study, we developed GAG microarrays for the GAG‐specificity screening of the SARS‐CoV‐2 S protein and demonstrated that the SARS‐CoV‐2 S protein binds not only to HEP/HS but also to CSE (Fig. 3). The S protein exhibited binding signals to CSE that were similar to binding signals to HS (Fig. 3). The binding of the S protein to HS2–BSA was inhibited by CSE and HEP/HS (HS2 and HS3; Fig. 4). This result is consistent with the previous study in which the binding of S protein to HEP is inhibited by not only HEP and HS, but also CSE [8]. CS, a disaccharide repeating unit of GalNAcβ1‐4GlcA with modifications of sulfate groups, is a GAG found in all animals that exhibit essential physiological functions [9]. CSE is 4,6‐disulfated at GalNAc residue, and its relationship with viral infection has been indicated [31]. CSE has been reported to be attached on proteoglycans expressed on the surface of vascular endothelial cells, such as endocan [32], thrombomodulin [33], and syndecan‐1/4 [34]. CSE acts as an important component of the CS receptor for HSV [31]. In addition, CSE also inhibits the cellular infection of HSV [31]. Since CSE inhibited binding of the SARS‐CoV‐2 S protein to HS (Fig. 4), CSE might act as an alternative factor for HEP/HS in SARS‐CoV‐2 infection [5].

Interestingly, the S2 subunit was found to show binding activity to GAGs as well (Table S2) [5, 8, 19, 20]. While the S1 subunit bound specifically to HEP‐BSA, the S2 subunit alone and the S protein showed remarkable binding to HEP/HS‐BSA and CSE‐BSA (Fig. 3). Recently, the SARS‐CoV‐2 S protein was demonstrated to bind with a much higher affinity to HEP (Kd = 55 nm) compared with the RBD (Kd = 1 µm) alone [19], indicating the involvement of the S2 subunit for the interaction with GAGs. The S2 subunit might be involved in the binding of the SARS‐CoV‐2 S protein to GAGs by stabilizing the structure of the S protein and/or having direct interaction with GAGs. The binding ability of the S2 subunit to GAGs might be involved in the fusion of SARS‐CoV‐2 with the host cell membrane [6]. Further studies are needed to clarify the mechanism and function of the GAG‐binding activity of the S2 subunit in SARS‐CoV‐2 infection.

Microarray‐based methods have been developed for high‐throughput analysis of GAG‐binding specificity of proteins. GAGs from natural products with or without modifications and chemically or chemoenzymatically synthesized oligosaccharides were immobilized on a microarray platform and were used for GAG‐specificity analysis [17, 19, 35, 36, 37]. Recently, Liu *et al*. analyzed the detailed HS‐binding specificity of the SARS‐CoV‐2 S protein by using microarrays containing 80 synthetic HS oligosaccharides and identified hexa‐ and octa‐saccharides composed of IdoA2S‐GlcNS6S repeating units as optimal ligands for the S protein [19].

In this study, we built upon a previously developed glycoconjugate microarray that was based on a fluorescence detection system for GAG‐specificity analysis of the SARS‐CoV‐2 S proteins [15]. We found that the S protein binds to CSE and HEP/HS. S1 bound specifically to HEP, whereas S2 showed binding activity to CSE and HEP/HS. Therefore, S2 might contribute substantially to the binding of the S protein to GAG expressed on host cells. Whether CSE acts as an attachment factor in SARS‐CoV‐2 infection awaits further evaluation. The binding activity of the S2 subunit to GAG should extend understanding of the mechanism underlying SARS‐CoV‐2 infection.

## Author contributions

TW wrote the paper. KT and KH performed experiments and analyzed the data. TM supervised the research. HT analyzed the data and wrote the paper.

## Funding source

This research was partially supported by Yakult Bio‐Science Foundation.

## Supporting information


**Fig. S1.** Screening of the GAG‐binding specificity of galectins.

**Table S1.** GAG‐BSA used in this study.

**Table S2.** Binding analysis between SARS‐CoV‐2 S protein and GAGs reported in previous studies.

## Data Availability

The data that support the findings of this study are available from the corresponding author [h-tateno@aist.go.jp] upon reasonable request.
