# Supplementary material for: A glycosaminoglycan microarray identifies the binding of SARS‐CoV‐2 spike protein to chondroitin sulfate E
Source: FEBS Lett. 2021 Aug 17;595(18):2341–9. doi: 10.1002/1873-3468.14173 (PMC8427098; doi:10.1002/1873-3468.14173)
Supplement: Supplementary file 1 — Fig. S1. Screening of the GAG‐binding specificity of galectins. [file FEB2-595-2341-s002.pdf]

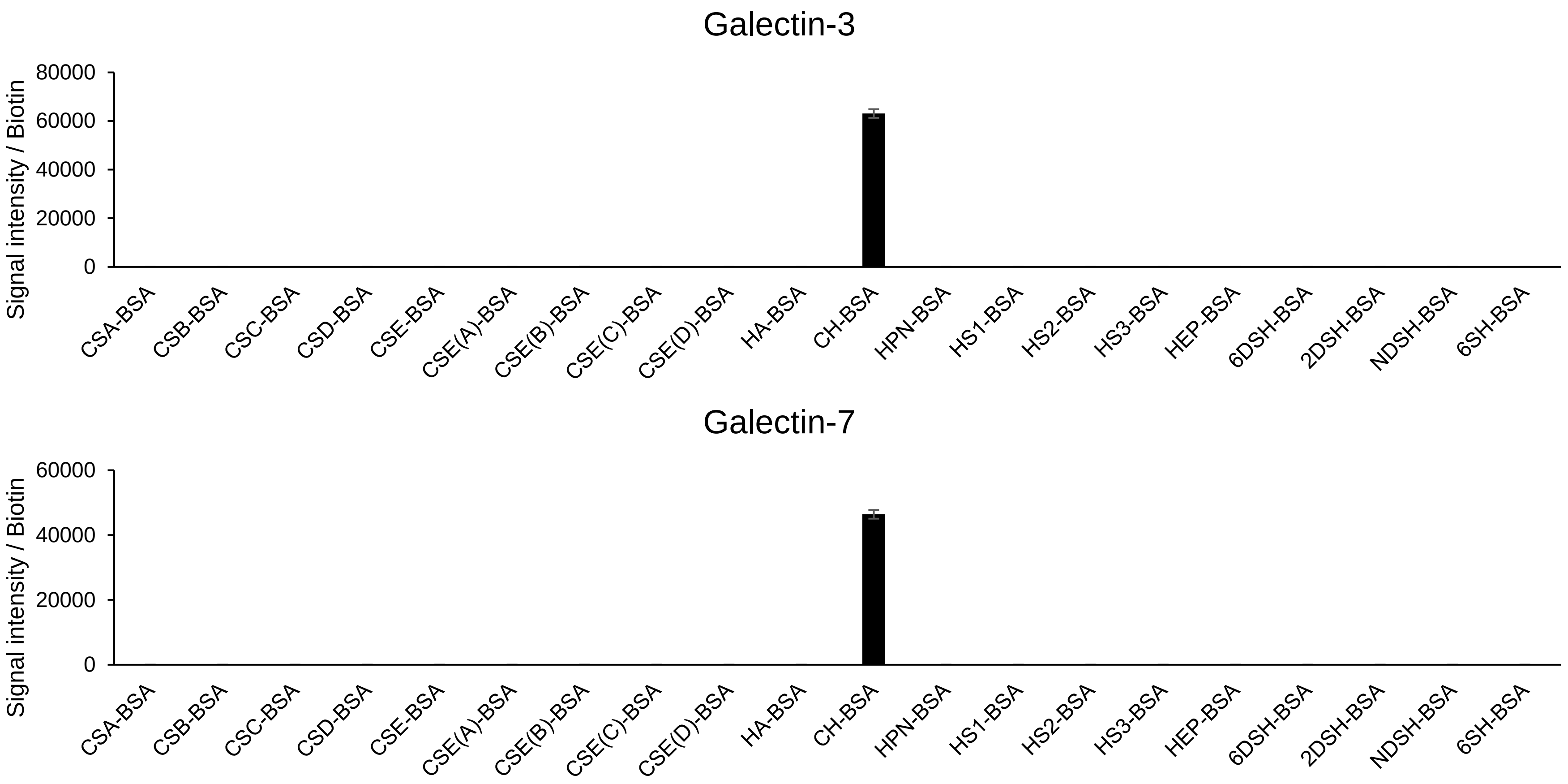

Fig. S1. Screening of the GAG-binding specificity of galectins. GAG microarrays were incubated with 10 μg/mL of galectin-3 (C-terminal carbohydrate-recognition domain, 108-250 aa, P17931) and galectin-7 (PDB: 8-136 aa, P47929) and were detected by the scanner without washing. Signal intensity was divided by the amount of biotin. Data are the average ± S.D. of triplicate spots.
