# Supplementary material for: A glycosaminoglycan microarray identifies the binding of SARS‐CoV‐2 spike protein to chondroitin sulfate E
Source: FEBS Lett. 2021 Aug 17;595(18):2341–9. doi: 10.1002/1873-3468.14173 (PMC8427098; doi:10.1002/1873-3468.14173)
Supplement: Supplementary file 2 — Table S1. GAG‐BSA used in this study. [file FEB2-595-2341-s001.pdf]

Table S1. GAG-BSA used in this study

	GAG source	Manufacturer	Typical disaccharide repeat of GAG	MW (kDa)	Biotin (nmol/mg)	Disaccharide composition analysis (%)								S content	Immobilization conc. (mg/mL)
						ΔDi-0S	ΔDi-4S	ΔDi-6S	ΔDi-U2S	ΔDi-diSE (4,6) S	ΔDi-diSB (U2,4) S	ΔDi-diSD (U2,6) S	ΔDi-triS		
CSA-BSA	Whale cartilage	Seikagaku	GlcAβ1-3GalNAc4S	25.4	12.8	1.8	75.2	23.0	0.0	0.0	0.0	0.0	0.0	6.8%	1
CSB-BSA	Pig skin	Seikagaku	IdoAα1-3GalNAc4S	22.8	6.5	0.4	91.4	1.6	0.0	0.0	6.6	0.0	0.0	7.5%	1
CSC-BSA	Shark cartilage	Seikagaku	GlcAβ1-3GalNAc6S	60.4	11.1	1.4	16.7	74.4	0.0	0.8	0.0	6.8	0.0	7.6%	1
CSD-BSA	Squid cartilage	Seikagaku	GlcA2Sβ1-3GalNAc6S	42.6	10.5	1.5	40.7	36.4	0.0	0.7	0.0	20.7	0.0	8.2%	0.3
CSE-BSA	Squid cartilage	Seikagaku	GlcAβ1-3GalNAc4,6diS	98.9	11.2	9.4	28.8	7.1	0.0	54.6	0.0	0.0	0.0	10.1%	0.23
CSE(A)-BSA	Squid cartilage	Seikagaku	GlcAβ1-3GalNAc4,6diS	46.7	9.9	10.2	25.7	8.9	0.0	47.7	0.0	0.0	0.0	10.4%	0.08
CSE(B)-BSA	Squid cartilage	Seikagaku	GlcAβ1-3GalNAc4,6diS	26.0	11.8	11.6	25.2	14.4	0.0	42.5	0.0	0.0	0.0	9.9%	0.15
CSE(C)-BSA	Squid cartilage	Seikagaku	GlcAβ1-3GalNAc4,6diS	35.1	11.0	10.5	25.3	11.5	0.0	46.1	0.0	0.0	0.0	9.6%	0.09
CSE(D)-BSA	Squid cartilage	Seikagaku	GlcAb1-3GalNAc4,6diS	17.5	10.7	14.0	23.9	18.4	0.0	37.8	0.0	0.0	0.0	9.0%	0.06
HA-BSA	Rooster combs	Seikagaku	GlcAβ1-3GlcNAc	35.9	6.6	100.0	0.0	0.0	0.0	0.0	0.0	0.0	0.0	0.0%	1
CH-BSA	Recombinant *Escherichia coli* fermentation	prepared in this study	GlcAβ1-3GalNAc	24.1	5.4	100.0	0.0	0.0	0.0	0.0	0.0	0.0	0.0	0.0%	1
HPN-BSA	Recombinant *Escherichia coli* fermentation	prepared in this study	GlcAβ1-3GlcNAc	41.1	5.8	100.0	0.0	0.0	0.0	0.0	0.0	0.0	0.0	0.0%	1
HS1-BSA	Pig kidney	prepared in this study	IdoA2Sβ1-4Glc6SNS	11.6	4.3	73.4	15.3	5.1	0.0	2.1	4.1	0.0	0.0	4.5%	0.65
HS2-BSA	Pig kidney	prepared in this study	IdoA2Sβ1-4Glc6SNS	26.7	10.6	32.0	16.0	22.2	0.0	16.6	3.4	0.0	9.7	7.7%	0.15
HS3-BSA	Pig kidney	prepared in this study	IdoA2Sβ1-4Glc6SNS	32.6	11.2	42.9	21.7	17.5	0.0	8.4	5.7	0.0	4.0	6.5%	0.18
HEP-BSA	Porcine intestinal Mucosa	Wako	IdoA2Sβ1-4Glc6SNS	15.4	9.5	7.0	4.6	4.4	1.5	11.0	8.0	3.5	60.0	13.2%	0.5
6DSH-BSA	Porcine intestinal Mucosa	Wako	IdoA2Sβ1-4GlcNS	14.5	9.7	14.3	21.3	0.0	3.0	2.2	59.2	0.0	0.0	10.8%	0.34
2DSH-BSA	Porcine intestinal Mucosa	Wako	IdoAβ1-4Glc6SNS	11.3	11.2	5.9	12.0	0.0	0.0	82.1	0.0	0.0	0.0	11.8%	0.27
NDSH-BSA	Porcine intestinal Mucosa	Wako	IdoA2Sβ1-4Glc6S	13.9	13.5	5.1	0.0	11.9	4.5	0.8	0.0	77.0	0.7	11.0%	1
6SH-BSA	Porcine intestinal Mucosa	Wako	IdoAβ1-4Glc6S	15.9	11.8	18.7	1.1	74.6	0.8	0.6	0.0	4.2	0.0	6.5%	1
