# Supplementary material for: A glycosaminoglycan microarray identifies the binding of SARS‐CoV‐2 spike protein to chondroitin sulfate E
Source: FEBS Lett. 2021 Aug 17;595(18):2341–9. doi: 10.1002/1873-3468.14173 (PMC8427098; doi:10.1002/1873-3468.14173)
Supplement: Supplementary file 3 — Table S2. Binding analysis between SARS‐CoV‐2 S protein and GAGs reported in previous studies. [file FEB2-595-2341-s003.pdf]

Table S2. Binding analysis between SARS-CoV-2 S protein and GAGs reported in previous studies

S protein	GAG	Method	Reference
Trimeric S protein, trimeric S1 RBD protein, SARS-CoV-2 virus	HEP, HS	ELISA, flow cytometry	Clausen et al. (2020)
Monomeric and trimeric S protein	HEP	SPR	Kim et al. (2020)
Pseudoviral particles bearing SARS-CoV-2 S protein	HEP, HS, CS	Pulldown assay, viral entry assay	Zhang et al. (2020)
S1 RBD protein, monomeric and trimeric S protein	HEP, HS	SPR	Liu et al. (2020)
S1 RBD protein, SARS-CoV-2 virus	HEP, HS	SPR, viral entry assay	Mycroft-West et al. (2020)
S protein, S1 RBD protein	3-O-sulfated HS	Cell-to-cell fusion model	Tiwari et al. (2020)
SARS-CoV-2 virus	HEP, Tinzaparin, Dalteparin	Viral entry assay	Tree et al. (2020)
S protein pseudotyped on a lentiviral vector	HEP, NDS-HEP, OS-HEP, 2DS-HEP, 6DS-HEP	Flow cytometry, cell-based ELISA, SPR	Yue et al. (2021)
S protein, S1 protein, S1 RBD protein	HEP, Enoxaparin, Deltaparin, Fondaparinux	Flow cytometry	Partridge et al. (2021)
S protein pseudotyped on a lentiviral vector	HEP, HEP-de6S, Enoxaparin, Enoxaparin-de6S, sulfated fucan, sulfated galactan, HS, CSA, DS, CSC, CSD, CSE, KS, Arixtra	SPR, viral entry assay	Tandon et al. (2021)
Trimeric S protein	HEP, HS	MD simulation	Schuurs et al. (2021)
Monomeric and trimeric S protein	HS	HS microarrays, SPR	Hao et al. (2021)
